# Fibroblast Growth Factor 21 Ameliorates Na_V_1.5 and Kir2.1 Channel Dysregulation in Human AC16 Cardiomyocytes

**DOI:** 10.3389/fphar.2021.715466

**Published:** 2021-09-22

**Authors:** Jiamin Li, Yuanshi Li, Yining Liu, Hang Yu, Ning Xu, Di Huang, Yadong Xue, Sijia Li, Haixin Chen, Jiali Liu, Qingsui Li, Yiming Zhao, Ronghao Zhang, Hongru Xue, Yuehang Sun, Ming Li, Pengyu Li, Mingbin Liu, Zhen Zhang, Xin Li, Weijie Du, Ning Wang, Baofeng Yang

**Affiliations:** ^1^ The Department of Pharmacology and State-Province Key Laboratories of Biomedicine-Pharmaceutics of China, College of Pharmacy, Harbin Medical University, Harbin, China; ^2^ Key Laboratory of Cardiovascular Medicine Research, Ministry of Education, College of Pharmacy, Harbin Medical University, Harbin, China; ^3^ Department of Cardiology, The First Affiliated Hospital of Harbin Medical University, Harbin, China

**Keywords:** myocardial infarction, ventricular arrhythmias, ion channel, AC16, action potential duration, FGF21 (fibroblast growth factor 21)

## Abstract

Infarcted myocardium is predisposed to cause lethal ventricular arrhythmias that remain the main cause of death in patients suffering myocardial ischemia. Liver-derived fibroblast growth factor 21 (FGF21) is an endocrine regulator, which exerts metabolic actions by favoring glucose and lipids metabolism. Emerging evidence has shown a beneficial effect of FGF21 on cardiovascular diseases, but the role of FGF21 on ventricular arrhythmias following myocardial infarction (MI) in humans has never been addressed. This study was conducted to investigate the pharmacological effects of FGF21 on cardiomyocytes after MI in humans. Patients with arrhythmia in acute MI and healthy volunteers were enrolled in this study. Serum samples were collected from these subjects on day 1 and days 7–10 after the onset of MI for measuring FGF21 levels using ELISA. Here, we found that the serum level of FGF21 was significantly increased on day 1 after the onset of MI and it returned to normal on days 7–10, relative to the Control samples. In order to clarify the regulation of FGF21 on arrhythmia, two kinds of arrhythmia animal models were established in this study, including ischemic arrhythmia model (MI rat model) and nonischemic arrhythmia model (ouabain-induced guinea pig arrhythmia model). The results showed that the incidence and duration time of ischemic arrhythmias in rhbFGF21-treated MI rats were significantly reduced at different time point after MI compared with normal saline-treated MI rats. Moreover, the onset of the first ventricular arrhythmias was delayed and the numbers of VF and maintenance were attenuated by FGF21 compared to the rhbFGF21-untreated group in the ouabain model. Consistently, *in vitro* study also demonstrated that FGF21 administration was able to shorten action potential duration (APD) in hydrogen peroxide-treated AC16 cells. Mechanically, FGF21 can ameliorate the electrophysiological function of AC16 cells, which is characterized by rescuing the expression and dysfunction of cardiac sodium current (*I*_Na_) and inward rectifier potassium (*I*_k1_) in AC16 cells induced by hydrogen peroxide. Moreover, the restorative effect of FGF21 on Na_V_1.5 and Kir2.1 was eliminated when FGF receptors were inhibited. Collectively, FGF21 has the potential role of ameliorating transmembrane ion channels remodeling through the Na_V_1.5/Kir2.1 pathway by FGF receptors and thus reducing life-threatening postinfarcted arrhythmias, which provides new strategies for antiarrhythmic therapy in clinics.

## Introduction

Acute myocardial infarction (AMI) patients are prone to a variety of arrhythmias, among which ventricular arrhythmia has a higher incidence, and it is also one of the important factors leading to sudden death and endangering the lives of patients. In the United States, 80% of sudden deaths are caused by arrhythmia, and 20% of them have a history of myocardial infarction (MI) (Kallergis and Vardas, 2007). Mechanistically, abnormal electrophysiological changes including action potential (AP) prolongation and ion channel imbalance play critical roles in arrhythmia generation in the post-MI heart (Qin et al., 1996; Yang et al., 2015). Increasing evidence suggested that the imbalance of ion channel currents including Na^+^ current (Kelly et al., 2018), L-type Ca^2+^ current (Pessina et al., 2018), and various K^+^ currents (Decher et al., 2017; Ponce-Balbuena et al., 2018) leads to a prolongation of QT interval and contributes to serious ventricular arrhythmias in heart diseases (Al-Akchar and Siddique, 2017; Schwartz et al., 2020). The prolongation of AP repolarization is a hallmark of cardiomyocytes isolated from MI hearts and might lead to the dispersion of repolarization and development of after-depolarization (Tan et al., 1995). It has been reported that the main voltage-gated sodium channel expressed in cardiomyocytes is Na_V_1.5, and dysfunction of Na_V_1.5 will cause changes in the flux and duration of sodium current *I*_Na_, leading to various types of arrhythmias (Makara et al., 2014; Wei et al., 2016). In addition, *I*_K1_ mainly forms the repolarizing current during the terminal phase of the AP, thus serving as the primary conductance controlling the resting membrane potential (RMP) in ventricular myocytes, and ventricular myocytes from the border zone of MI hearts undergo a significant reduction of potassium channels in both its activity and expression in mouse, rat, and human beings (Liao et al., 2013; Ponce-Balbuena et al., 2018). However, how to rescue the dysfunction of ion channel and prevent sudden cardiac death in heart diseases remains to be resolved.

Human fibroblast growth factors (FGFs) are heparin-binding proteins that contain 23 members which can be divided into seven subfamilies based on phylogeny and sequence, which makes up a cytokine superfamily that regulates a plethora of developmental processes, including regulating mitosis, differentiation, development, metabolism, and angiogenic therapeutic applications (Beenken and Mohammadi, 2009; Itoh et al., 2016b). These functions are mediated, in part, by the interaction of FGFs with relatively high-affinity cell-surface receptors, microRNAs (miRNAs), and subsequent alterations in gene expression within responsive cells. FGF21 is a potent insulin-like growth factor that promotes energy expenditure, lipid excretion, and fat utilization (Cheng et al., 2016; Maratos-Flier, 2017). β-Klotho functions as an essential cofactor for FGF21 activity (Yie et al., 2012). Recent studies have shown that FGF21 plays a vital role in the onset and development of cardiovascular diseases. Ferrer-Curriu G et al. (2019) suggested that FGF21 acts as a key factor in the fibrogenesis associated with increased expression levels of β-klotho specifically in the heart (Ferrer-Curriu et al., 2019). In addition, Lin et al. showed that the high level of FGF21 may be associated with poor lipid level in patients with coronary heart disease (Lin et al., 2010). Our previous study uncovered for the first time that FGF21 treatment markedly reduced arrhythmia susceptibility of infarcted hearts in mice by suppressing miR-143 expression and thus mediating the EGR1-SCN5A/KCNJ2 pathway in MI mice (Li et al., 2020). Nevertheless, it remains unclear whether FGF21 has preventive effects on ischemic arrhythmias in humans.

To shed light on this issue, we explored the effect of FGF21 on the ion pathways of myocardial cells after MI and discovered the mechanism of preventing arrhythmia after MI by affecting the ion pathways. Our results unraveled for the first time that FGF21 protects Na_V_1.5 and Kir2.1 channels by receptors against arrhythmias after MI in humans.

## Materials and Methods

### Human Samples

According to the approval of the Harbin Medical University Ethics Committee (HMUIRB20170020), 11 cases were enrolled in the final analysis. Peripheral blood was obtained from eight patients undergoing AMI and three healthy volunteers. AMI group included AMI patients within 24 h after postinfarct onset. Chronic myocardial ischemia (CMI) group included patients with 7th-10th days after postinfarct onset. Healthy volunteers were used as normal control.

### Animals

Male guinea pigs (220–250 g) were housed under standard animal room conditions (temperature of 22 ± 1°C; humidity of 55–60%). Animals were randomly divided into three groups: Ctl, ouabain, and ouabain + rhbFGF21. The guinea pigs were anesthetized with isoflurane 2–4% (MIDMARK, USA) using a gas chamber. The animals were fixed to the mouse board in a supine position; three different concentrations of rhbFGF21 (1 μg/kg, 5 μg/kg, and 10 μg/kg) were pretreated at 2 h before 0.2 mg/ml ouabain at the same speed (1,000 μl/h) was injected into the external jugular vein of guinea pigs. Standard lead II electrocardiogram (ECG) signals were recorded using bipolar limb leads. Ventricular arrhythmia occurred for the first time, arrhythmia occurs at the time of death, and the arrhythmia numbers were detected by the biological signal analytical system (model BL-420N, TaiMeng Software). The investigation conforms to the Guide for the Care and Use of Laboratory Animals published by the US National Institutes of Health (NIH Publication No. 85–23, revised 1996) and was approved by the Institutional Animal Care and Use Committee of Harbin Medical University.

Male SD rats (180–220 g) were housed under standard animal room conditions (temperature of 22 ± 1°C; humidity of 55–60%). SD rats were randomly divided into two groups: sham and MI groups. The operation of left anterior-descending branch (LAD) coronary artery ligation was performed in MI rats, as previously described (Lindsey et al., 2018). ST-segment elevation in ECGs indicates successful occlusion of the artery. Sham group rats served as surgical controls and were subjected to the same procedures as MI group rats, except the LAD ligation.

### Enzyme-Linked Immunosorbent Assay

Human and rat blood serum were collected for measurement of FGF21 concentrations using an ELISA kit (human, Elabscience Biotechnology Co., Ltd.; Catalog No: E-EL-H0074c, and rat, Elabscience Biotechnology Co., Ltd.; Catalog No: E-EL-R2408c), according to the manufacturer’s instructions with a lower limit of detection of 18.75 pg/ml. Allow samples for 20 min at room temperature before centrifugation at approximately 1,000 g. Add 100 µl of standard, blank, or sample per well and incubate for 90 min at 37°C; add 100 µl Biotinylated Detection Ab working solution and incubate for 1 h at 37°C; and add HRP Conjugate working solution and incubate for 30 min at 37°C. The level of FGF21 was recognized by a biotinylated detection antibody specific for FGF21 and Avidin Horseradish Peroxidase (HRP) conjugate. The optical density (OD) was measured spectrophotometrically at a wavelength of 450 ± 2 nm and finally analyzed according to the instructions of the kits.

### Whole-Cell Patch-Clamp Recording

Whole-cell patch-clamp recording techniques were used to record Na^+^ currents, K^+^ currents, and action potential duration (APD) in AC16 cardiomyocytes as described previously (Ennis et al., 2002; Kao et al., 2016; Li et al., 2020). *I*_Na_ recordings were conducted at room temperature (22–24°C); *I*_K1_ and APD recordings were conducted at 37°C using a patch-clamp amplifier (MultiClamp 700B; Axon Instruments Inc. Foster City, CA, United States). APs were recorded in the current-clamp mode by injection of brief current pulses (2 ms; 1 nA) at a stimulation frequency of 1 Hz; the area under the capacitance current curve was divided by the applied voltage step to obtain the total battery capacitance. AP duration at 50% and 90% amplitude repolarization was measured as APD_50_ and APD_90_, respectively. AP amplitude (APA) is obtained from the RMP to the peak of AP depolarization.

The ionic currents were measured in the voltage-clamp mode. Electrode resistance ranged from 2.0 to 3.0 MΩ. The isolated cells were placed in an inverted microscope (IX-70; Olympus) and infused with Tyrode solution. The bath solution for Na + currents contained (in mM) 145 NaCl, 4.5 CsCl, 1.5 MgCl_2_, one CaCl_2_, 5 HEPES, 5 glucose, and 0.1 CdCl_2_ (pH 7.35 with CsOH); the pipette solution contained (in mM) 10 NaF, 110 CsF, 20 CsCl, 10 EGTA, and 10 HEPES (pH 7.35 with CsOH). The external solution for recording K^+^ currents contained (in mM) 136 NaCl, 5.4 KCl, 0.33 NAH_2_PO_4_, one MgCl_2_.6H_2_O, 1.8 CaCl2, 5 HEPES, and 10 glucose (pH 7.37 with NaOH). The pipette solution for recording K+ currents contained (in mM) 20 KCl, 110 L-Aspartate, 5 HEPES, one MgCl_2_, 5 Na_2_ATP, and 10 EGTA (pH 7.20 with KOH). The experiments were controlled by using pClampex 10.6 software (Axon Instruments Inc., Foster City, CA, United States). Currents were acquired by Digidata 1440A converter (Axon Instruments Inc., Foster City, CA, United States), low-pass filtered at 5 kHz, and stored on a computer. A single current was standardized to membrane capacity to control cell size differences and expressed as current density (pA/pF). Data analysis was accomplished using pClampfit 10.4 software (Axon Instruments Inc., Foster City, CA, United States).

### Western Blot Analysis

Western blot analysis was performed on total proteins extracted from AC16 cells. Sixty milligrams of protein was equally loaded on 10% SDS-polyacrylamide gel and then transferred onto a nitrocellulose filter membrane. After getting the target bands, the membranes were blocked by 5% filtered nonfat milk for 2 h and then incubated with the indicated primary antibodies overnight at 4°C, including rabbit anti-Na_V_1.5 antibody (ASC-005, Alomone Labs, Israel, 1: 200), rabbit anti-Kir2.1 antibody (APC-026, Alomone Labs, Israel, 1: 200) followed by incubation with the corresponding secondary antibody for 1 h at room temperature. Protein levels were quantified using the Odyssey Infrared Imaging System (LI-COR, 4607 Superior Street, P.O. Box 4,000, Lincoln, Nebraska, United States) by measuring proteins’ gray values and the data was normalized to β-actin (TA-09, Zhongshanjinqiao, Beijing, China, 1:1,000) as an internal control.

### Immunofluorescence

AC16 cells were fixed in 4% paraformaldehyde (PFA) and permeabilized with 0.4% Triton-X at room temperature for 20 min. The samples were coincubated with Na_V_1.5 (ASC-005, Alomone Labs, Israel, 1:200), Kir2.1 (APC-026, Alomone Labs, Israel, 1: 200), and α-actinin (ab50599, Abcam, Cambridge, United Kingdom, 1:100) antibody at 4°C overnight. Then the AC16 cells were incubated with the appropriate secondary antibody at room temperature for 2 h and the nuclei were stained with DAPI (Beyotime, Shanghai, China, 1:1,000) for 10 min. Immunofluorescence was observed under a confocal laser scanning microscope (Nikon 80i, Otawara, Tochigi, Japan).

### Statistical Analysis

Data are presented as mean ± SEM. Two-tailed Student’s *t*-test and ANOVA were used to compare the difference among each group. *p*-value less than 0.05 was defined as statistical significance. All statistical analyses were carried out using GraphPad Prism 8.0 (GraphPad Software Inc., San Diego, CA, United States).

## Results

### Elevation of Serum FGF21 Level in Patients and Rats With MI

**FIGURE 1:**
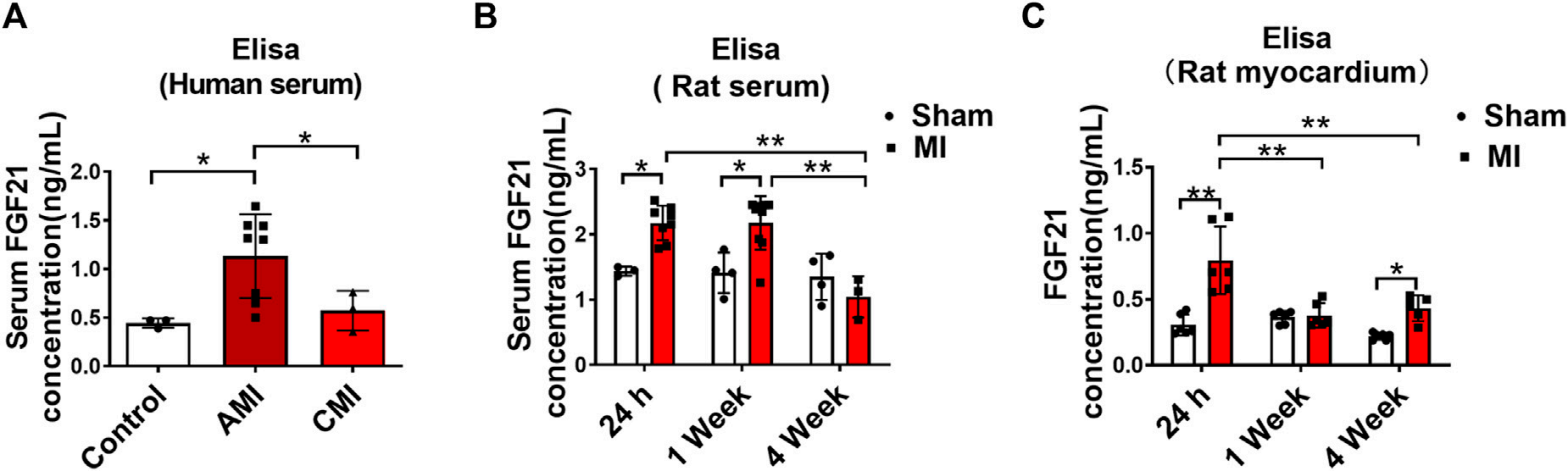
Serum FGF21 levels in patients and rats with myocardial infarction. **(A)** The FGF21 levels in peripheral blood serum from healthy volunteers (Control, *n* = 3) and myocardial infarction (MI) subjects (*n* = 8) on the first day (AMI) and 7th-10th day (CMI, *n* = 3). Two-tailed Student’s *t*-test was performed, *p < 0.05. **(B)** Comparison of serum FGF21 levels in rats between MI and sham operation group (sham). *n* = 3–7, *p < 0.05 and **p < 0.01. **(C)** Comparison of FGF21 levels in rat heart tissues between MI and sham group. *n* = 6, *p < 0.05 and **p < 0.01.

Patients with arrhythmia in acute MI and healthy volunteers were enrolled in this study. Serum samples were collected from these subjects on day 1 and days 7–10 after the onset of MI for measuring FGF21 levels using ELISA. The results showed that the serum level of FGF21 was significantly increased on day 1 after the onset of MI and it returned to normal on days 7–10, relative to the Control samples (Figure 1A). In our rat model of MI, the serum FGF21 level reached the maximum value within approximately 24 h (acute MI phase) after the onset of MI. Thereafter, from 1 week to 4 weeks after MI, it began to decrease compared with the acute MI phase, but it remained high for 1 week (Figure 1B). The elevation in MI serum does not necessarily mean that the FGF21 concentration is increased in the interstitial space within the heart; the results in Figure 1C showed that the level of FGF21 in heart tissue homogenate was significantly increased in the acute MI phase, which began to decrease in chronic MI period, relative to acute MI phase. It raises the possibility that FGF21 might be involved in regulating the pathological process of MI.

### FGF21 Administration Can Reduce Ventricular Arrhythmia in MI Model and Ouabain Model

Therefore, in order to ascertain whether FGF21 exerts a protective effect on ischemic arrhythmia, we administered FGF21 to MI model rats and recorded the electrocardiogram of each group in the previous study. The results showed that the duration time of ventricular arrhythmias in rhbFGF21-treated MI rats was significantly reduced at 15 min, 1 week, and 4 weeks after MI compared with normal saline-treated MI rats. Moreover, the incidence of ventricular arrhythmias was also obviously decreased in the rhbFGF21-treated MI rats as compared to the MI group (Li et al., 2020). These data suggested that FGF21 has a protective effect on ischemic arrhythmias after MI.

Secondly, we used ouabain-induced arrhythmia of the guinea pig model, a classic nonischemic arrhythmia model, to further explore whether FGF21 has a regulatory effect on malignant ventricular arrhythmia and sudden cardiac death. We investigated that the onset of the first ventricular arrhythmias (arrhythmia in incubation period) was delayed by FGF21 compared to the rhbFGF21-untreated group in the ouabain model **(** Supplementary Figures S1A,B **)**. At the same time, it was found that the survival time of guinea pigs induced by ouabain can be obviously ameliorated with the increase of FGF21 concentration **(** Supplementary Figure S1C **)**. In addition, the numbers of VF and maintenance were attenuated by rhbFGF21 pretreatment compared to the rhbFGF21-untreated group **(** Supplementary Figure S1D,E **)**, but these were not statistically significant.

### FGF21 Upregulates *I*_Na_ and *I*_K1_ Channel in H_2_O_2_-Treated Cells

**FIGURE 2:**
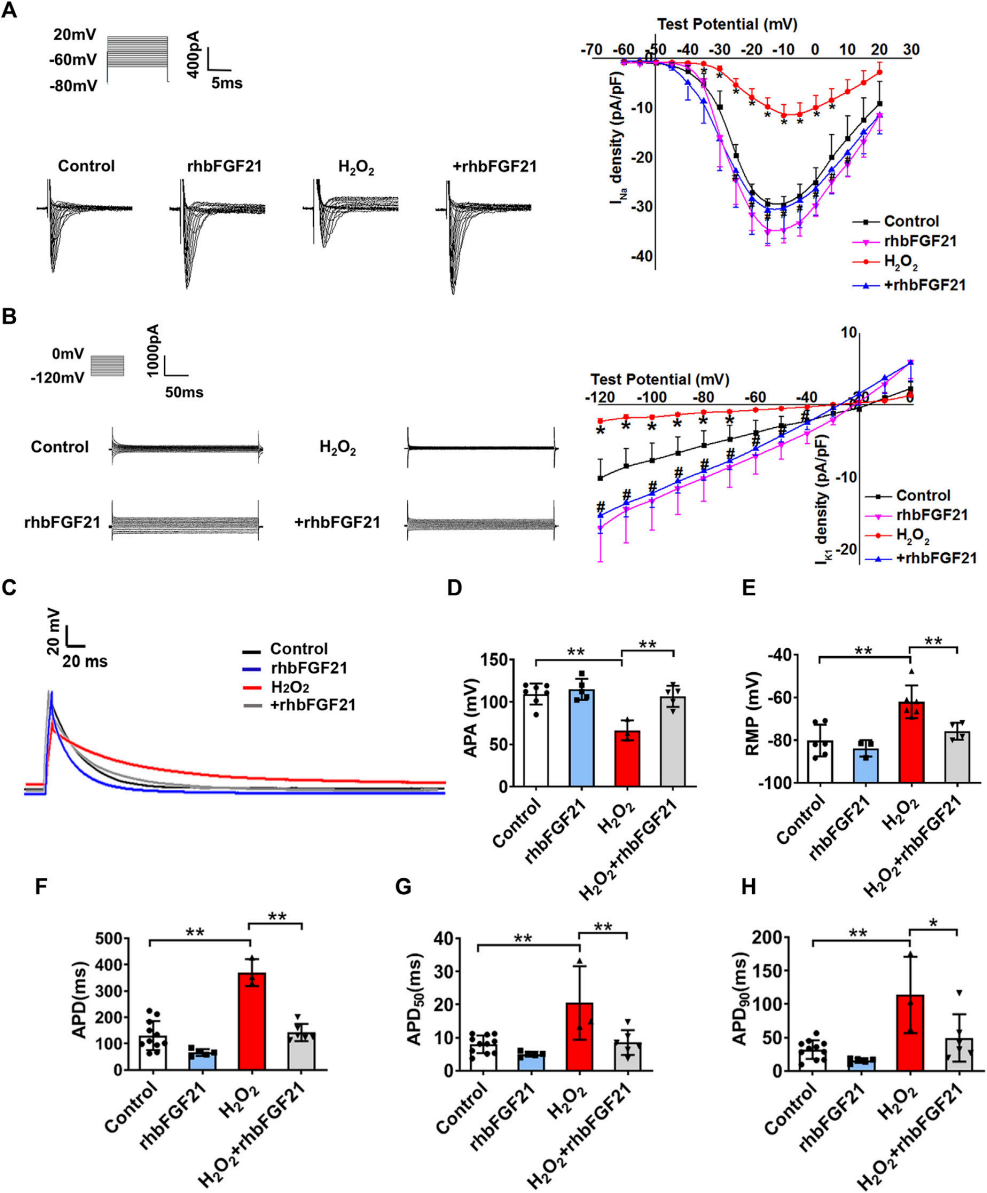
Effects of FGF21 on Na^+^ channel (*I*_Na_) and K^+^ channel (*I*_K1_) currents of AC16 human cardiomyocytes. **(A, B)** The current density-voltage relationships curves of whole-cell sodium current (*I*_Na_) and potassium current (*I*_K1_) were recorded in AC16 cells treatment with H_2_O_2_ and H_2_O_2_+rhbFGF21. *p < 0.05 vs. Control. **(C)** Exemplar superimposed traces of APD recorded from myocytes, and comparison of average data of APD **(F)**, APD_50_ **(G)**, and APD_90_ **(H)** measured AC16 human cardiomyocytes from Control, rhbFGF21, H_2_O_2_, and H_2_O_2_+rhbFGF21. One-way ANOVA with Tukey’s multiple comparisons test was used to compare the difference between multiple groups, *p < 0.05; **p < 0.01. **(D, E)** The APA and RMP of AC16 cells from Control, rhbFGF21, H_2_O_2_, and H_2_O_2_+rhbFGF21 group recorded by patch-clamp technique. *n* = 3–7, **p < 0.01.

To define the functional impact of FGF21 on the currents of Na_V_1.5 and Kir2.1, *I*_Na_ and *I*_K1_ were recorded using a whole-cell patch clamp. Figure 2A and Figure 2B display the representative current traces generated by *I*_Na_ and *I*_K1_ channels in AC16 cells treated with rhbFGF21 75 nM for 24 h with or without H_2_O_2_ treatment. We found that FGF21 restored the reduction of *I*_Na_ current density at the tested voltages from −25 to 10 mV compared with the H_2_O_2_ group, whereas there is no difference of *I*_Na_ density between Control and rhbFGF21-infected cells alone (Figure 2A). Moreover, at the test potentials of −120 mV to −40 mV, the densities of *I*_K1_ recorded in AC16 cells with FGF21 plus H_2_O_2_ treatment were significantly increased compared to that with H_2_O_2_ administration alone (Figure 2B). We also assessed the effect of FGF21 on the APD of AC16 cells treated with rhbFGF21 75 nM for 24 h with or without H_2_O_2_ treatment (Figure 2C). Figures 2D,E show that the action potential amplitude (APA) and the absolute value of RMP of H_2_O_2_ treatment cells decreased significantly, which were attenuated by rhbFGF21 administration. Moreover, the H_2_O_2_ treatment cells also displayed a significant prolongation of APD (∼182.81%, *p* < 0.01), APD_50_ (∼156.25%, *p* < 0.01), and APD_90_ (∼256.72%, *p* < 0.01). Nevertheless, rhbFGF21-treated cells showed shorter APD (∼61.47%, *p* < 0.01), APD_50_ (∼58.44%, *p* < 0.01), and APD_90_ (∼56.7%, *p* < 0.01) than those in H_2_O_2_ group, and no significant difference was found between Control and rhbFGF21 treatment alone (Figures 2F–H). These results indicate that FGF21 can restore the function of *I*_Na_ and *I*_K1_ currents and ameliorate the electrophysiological remodeling of cardiomyocytes induced by hydrogen peroxide.

### Potential Role of FGF21 in Na_V_1.5 and Kir2.1 *In Vitro*

**FIGURE 3:**
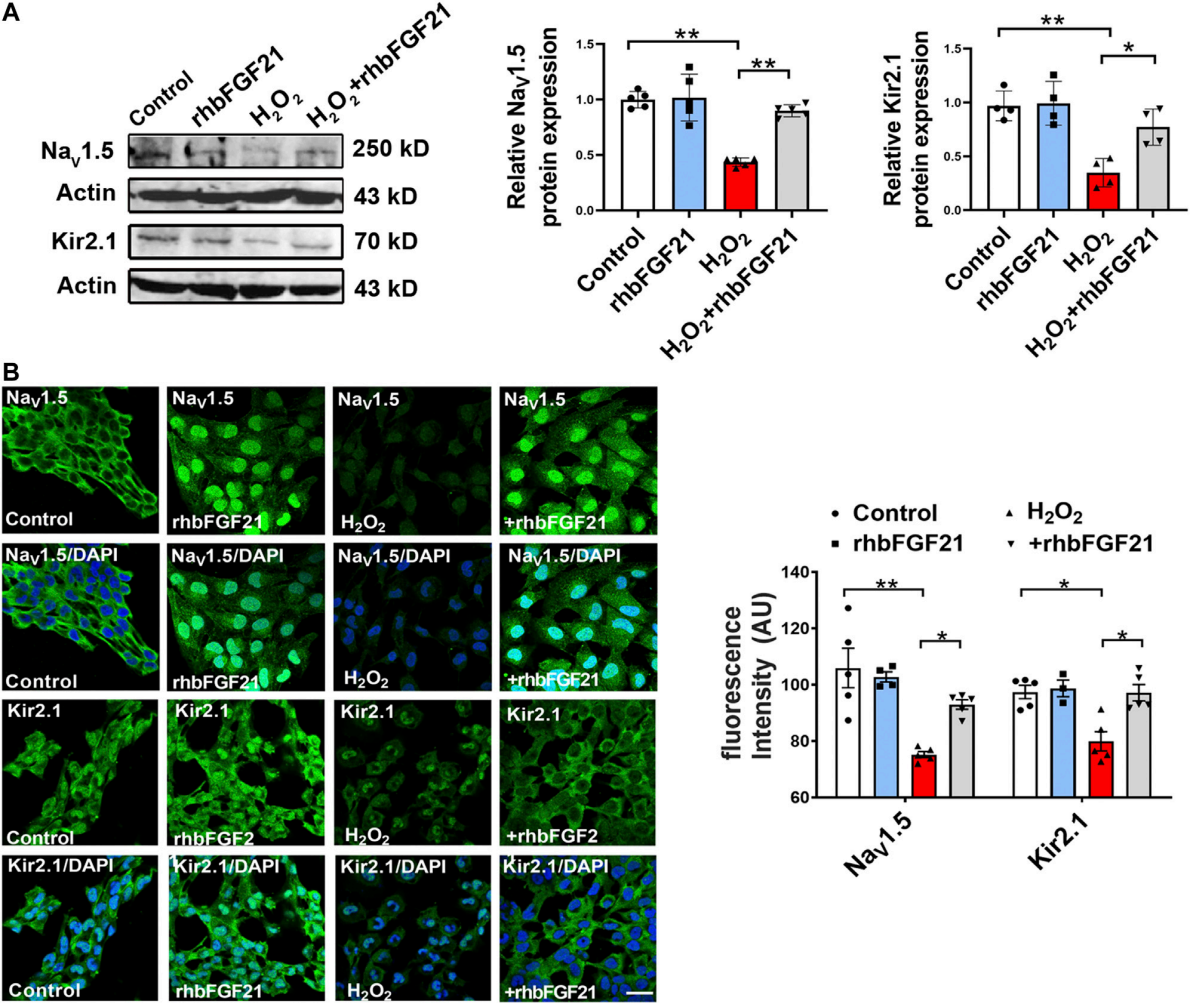
Effects of FGF21 on Na_V_1.5 and Kir2.1 expression in human AC16 cells. **(A)** Protein level of Na_V_1.5 and Kir2.1 in AC16 human cardiomyocytes treatment with H_2_O_2_ (100 μmol) for 6 h, *n* = 3–4 batches of cells, *p < 0.05; **p < 0.01. Analysis by one-way ANOVA, mean ± SEM. **(B)** Immunofluorescence staining of Na_V_1.5 and Kir2.1 expression in AC16 human cardiomyocytes treatment with H_2_O_2_. *p < 0.05; **p < 0.01. Analysis by one-way ANOVA, mean ± SEM. Scale bar indicates 100 μm.

To further clarify if the restoration of depressed *I*_Na_ and *I*_K1_ by rhbFGF21 is due to the upregulation of Na_V_1.5 and Kir2.1, we investigated the effects of FGF21 on the expression of Na_V_1.5 and Kir2.1 in the hydrogen peroxide-treated human AC16 cells using Western blot assay. As shown in Figure 3A, the treatment of rhbFGF21 elevated the Na_V_1.5 and Kir2.1 protein levels in H_2_O_2_-treated AC16 cells. Moreover, immunocytochemical staining proved the presence of Na_V_1.5 and Kir2.1 channels on the membrane of AC16 cells, and rhbFGF21 reversed the decrease of Na_V_1.5 and Kir2.1 protein expression by H_2_O_2_ treatment (Figure 3B).

### The Major Role of FGFR in Antiarrhythmia Effects of FGF21

**FIGURE 4:**
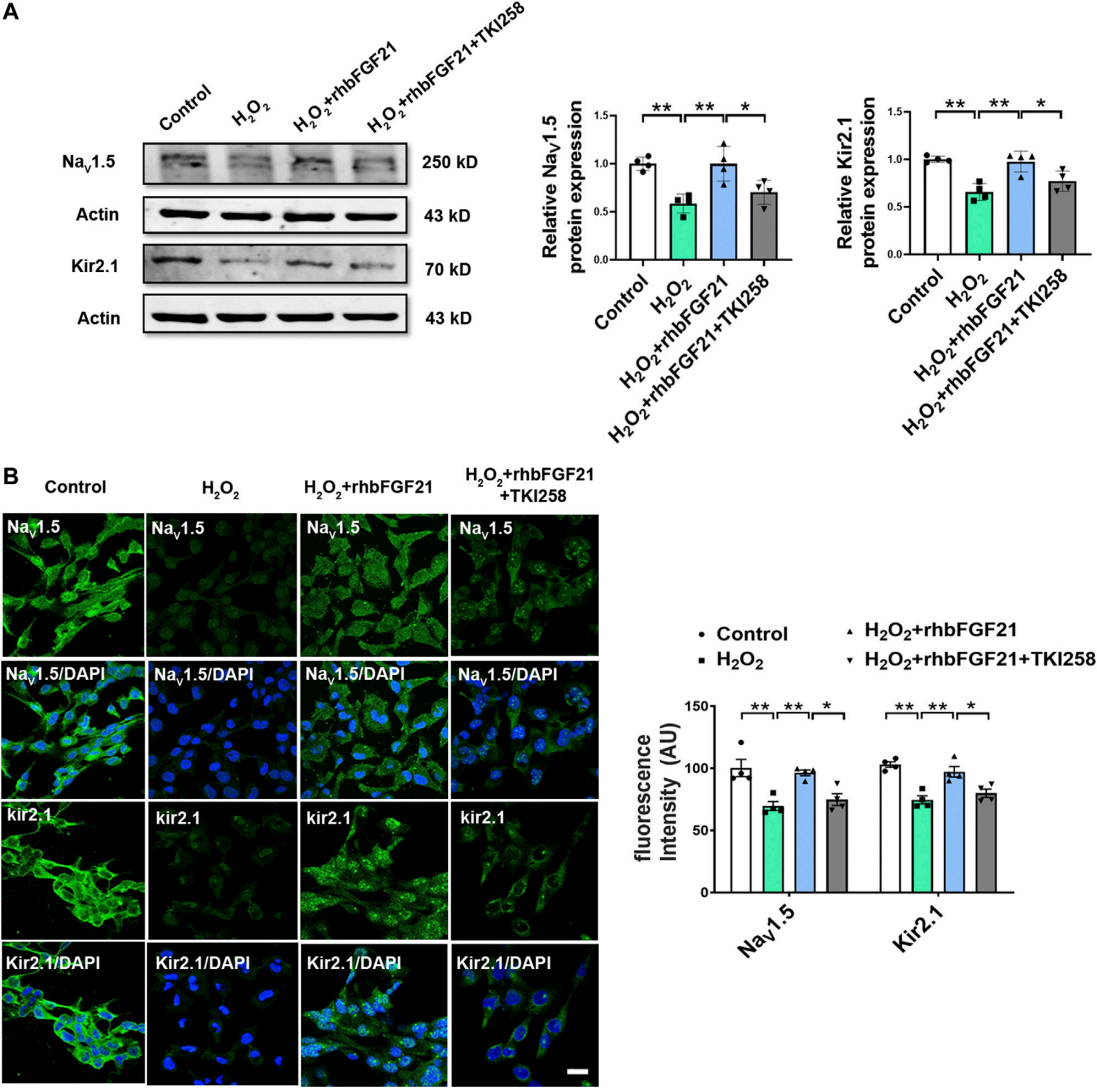
Effect of FGFR on Na_V_1.5 and Kir2.1 expression. **(A)** Protein level of Na_V_1.5 and Kir2.1 in AC16 human cardiomyocytes in Control, H_2_O_2_, H_2_O_2_+rhbFGF21, and H_2_O_2_+rhbFGF21 + TKI258 group, n = 4 batches of cells, *p < 0.05; **p < 0.01. Analysis by one-way ANOVA, mean ± SEM. **(B)** Immunofluorescence staining of Na_V_1.5 and Kir2.1 expression in AC16 human cardiomyocytes in each group. *p < 0.05; **p < 0.01. Analysis by one-way ANOVA, mean ± SEM. Scale bar indicates 100 μm.

Previous studies suggest that FGF21 binds FGF21 receptors (FGFR) for activating intracellular signaling pathways that ultimately generate biological effects. Consistently, our study showed that the expression of Na_V_1.5 and Kir2.1 in AC16 cardiomyocytes was obviously reduced after hydrogen peroxide treatment. Notably, in the presence of TKI258, FGF21 failed to increase the expression of Na_V_1.5 and Kir2.1 protein in AC16 cells significantly (Figure 4A). Similar effect of FGF21 was also observed in immunofluorescence staining (Figure 4B). It implies that FGF21 regulates electrophysiological properties of cardiomyocytes *via* FGFR-mediated Na_V_1.5 and Kir2.1 pathways.

## Discussion

In this study, we demonstrated for the first time that circulating levels of FGF21 rise substantially with MI. It is interesting that rhbFGF21 administration ameliorated the electrophysiological remodeling in H_2_O_2_-treated AC16 cells by increasing voltage-gated Na^+^ channel and K^+^ channel activity which is associated with FGFR-mediated upregulation of Na_V_1.5 and Kir2.1 expression and shortening of APD. The present study provides new insights into the role of FGF21 in postinfarct arrhythmias and suggests the potential application of FGF21 in the prevention of human cardiac sudden death.

Myocardium infarction was accompanied by the increased heterogeneity of conduction (Ng et al., 2016; Aronis et al., 2020; He et al., 2020), structural remodeling (Deniset et al., 2019), neurohumoral changes (Heeneman et al., 1997), alterations in transcription factor (Mccarroll et al., 2018), and ion channel protein expression and distribution (Speich et al., 1980), which alters cardiac electrical activity and often triggers malignant ventricular arrhythmias. Lots of experimental and clinical trials have reported that the incidence of ventricular arrhythmias and cardiac sudden death remains at high risk after MI (Pouleur et al., 2010). This highlights the need for further understanding of key mechanisms and development of innovative effective therapies for ventricular arrhythmias.

FGF21 is a typical endocrine signal, while function is a paracrine signal in hearts. Cardiac FGF21 secretion is obviously lower than hepatic FGF21 secretion (Itoh et al., 2016a). FGF21 is abundantly secreted by cardiac cells in response to cardiac stress, including cardiac hypertrophy, myocardial ischemia, heart failure, and diabetic cardiomyopathy (Planavila et al., 2015). Recently, increasing evidence has suggested that FGF21 plays a critical role in cardiovascular protection (Pan et al., 2018). However, the contribution of FGF21 to arrhythmias and electrical remodeling after MI in humans still remains unclear.

In order to clarify the regulation of FGF21 on arrhythmia, two kinds of arrhythmia animal models were established in our study, including ischemic arrhythmia model (MI rat model) and nonischemic arrhythmia model (ouabain-induced guinea pig arrhythmia model). Firstly, we administered rhbFGF21 to MI rats and recorded the electrocardiogram of each group in our previous study (Li et al., 2020). The results showed that the incidence and duration time of ischemic arrhythmias in rhbFGF21-treated MI rats were significantly reduced at different time point after MI. These data suggested that FGF21 has a protective effect on ischemic arrhythmias after MI. Based on the observation of ischemic arrhythmia in the MI rat model, we further clarify that the protective effect of FGF21 on arrhythmia depends on the pathological process of alleviating myocardial ischemia. We used ouabain-induced arrhythmia of the guinea pig model, a classic nonischemic arrhythmia model, to further explore the regulatory effect of FGF21 on arrhythmia. Ouabain is known as G-strophanthin, which belongs to cardiac glycoside drugs. Cardiac glycoside inhibits Na^+^-K^+^-ATP enzyme, thus increasing [Na]i and decreasing [K]i in cardiomyocytes and causing more Ca^2+^ in cardiomyocytes (Nosztray et al., 1989; Diederich et al., 2017). Ouabain-induced toxicity shows a variety of arrhythmias, including tachyarrhythmia, bradycardia, ventricular fibrillation, and even cardiac arrest, by causing dysfunction of ion channels in cardiomyocytes. We found that rhbFGF21 significantly prolonged the latency of ouabain-induced ventricular fibrillation and extend the survival time of guinea pigs with malignant arrhythmia. Therefore, we hypothesized that the protective effect of FGF21 on arrhythmia may be related to maintaining the functional balance of ion channels or stabilizing the myocardial cell membrane.

It has been reported that the inwardly rectifying potassium current (*I*_K1_) is considerable for maintenance of the cell RMP and modulating cardiomyocytes repolarization by altering transmembrane currents (Amoros et al., 2013; Klein et al., 2017; Ponce-Balbuena et al., 2018), and the voltage-gated Na + current (*I*_Na_) produces a rapid depolarizing current during the upstroke of the AP (Shy et al., 2013). Both a reducing potassium currents and an inappropriate delay in the entry of sodium currents into cardiomyocytes may contribute to the QT interval prolongation during the post-infarct ventricular arrhythmias (Goldenberg and Moss, 2017). Consistently with previous reports, our study showed that FGF21 restored the decreased *I*_K1_ currents and the decrease of *I*_Na_ currents in H_2_O_2_-treated AC16 cells compared to the normal group. Moreover, FGF21 treatment also caused larger APA of the AP and acceleration of cardiac depolarization in ventricular myocytes. In addition, the increasing P wave amplitude and the shortening of the QRS complex in surface ECGs were found in conscious rats pretreated with rhbFGF21. Moreover, we also investigated that the manipulation of *I*_K1_ density had a comparatively greater impact on APD_90_ because outward *I*_K1_ is larger at voltages in the vicinity of APD_90_. These results suggested that FGF21 plays a protective role in electrical disturbance H_2_O_2_-treated AC16 cells at least through restoring *I*_K1_ and *I*_Na_.

However, our study has limitations. *I*_K1_-mediated potassium ion balance is the main component of the RMP and we found that FGF21 can significantly restore the 4-phase RMP of AC16 cardiomyocytes, so in this study, we mainly tested the effect of FGF21 on the *I*_K1_ current. In addition, our results in human AC16 cardiomyocytes are consistent with previously published results which found that FGF21 can also significantly restore the *I*_K1_ current of isolated ventricular myocytes in mice (Li et al., 2020). *I*_Kr_ (rapidly activated delayed rectifier potassium current) and *I*_Ks_ (slowly activated delayed rectifier potassium current) cooperate with *I*_K1_ to mediate the process of cardiomyocyte repolarization. Because we did not functionally analyze *I*_Kr_ and *I*_Ks_ with patch clamp, we cannot exclude potential contribution of FGF21 function on other ion channels in our cell model which should be defined in future studies. In addition, through the patch-clamp technique, we found that FGF21 could restore the sodium current level of cardiomyocytes. However, the electrophysiological activities of cells are not only dependent on the expression level of ion channels but also closely related to their functions. In the process of detecting the APD, we found that FGF21 upregulated the resting potential of AC16 cardiomyocytes treated with hydrogen peroxide, as well as the levels of APA and Vmax. Sodium channels are voltage-gated ion channels which open when the membrane potential reaches a certain threshold. When the RMP decreases, it is difficult to stimulate the membrane potential to reach the threshold value of the sodium channel and the activity of the sodium channel will decrease accordingly. These results suggest that FGF21 may indirectly affect the inactivation status of sodium channels by ameliorating the changes of resting potential. Although our detection also found that FGF21 could restore the expression of Nav1.5 protein, the physiological correlation between FGF21 and the change of *I*_Na_ channel expression needs to be further explored.

Previous studies have shown that FGF21 binds to the FGF21 receptor (FGFR) to activate intracellular signaling pathways that ultimately produce biological effects (Zhao et al., 2019). Consistently, our study showed that TKI258, an FGFR antagonist, may decrease the expression of Na_V_1.5/Kir2.1 attenuated by FGF21 in AC16 cells. This means that FGF21 may regulate the electrophysiological properties of cardiomyocytes through FGFR. However, the deep mechanism by which FGF21 regulates the function of sodium and potassium ion channels through receptors and whether FGF21 regulates ion remodeling in AC16 cardiomyocytes through other pathways still need to be further explored.

## Conclusion

In summary, we discovered for the first time that FGF21 protects the Na_V_1.5 and Kir2.1 channels through FGFR and this may contribute to preventing arrhythmia after MI in humans. These findings provide evidence for the antiarrhythmic effect of FGF21 and reveal the potential application prospects of FGF21 in the treatment of arrhythmia after MI in humans.

## Data Availability

The datasets presented in this study can be found in online repositories. The names of the repository/repositories and accession number(s) can be found in the article/Supplementary Material.
