# Supplementary figures and images for: Fibroblast Growth Factor 21 Ameliorates NaV1.5 and Kir2.1 Channel Dysregulation in Human AC16 Cardiomyocytes
Source: Front Pharmacol. 2021 Sep 22;12:715466. doi: 10.3389/fphar.2021.715466 (PMC8493335; doi:10.3389/fphar.2021.715466)

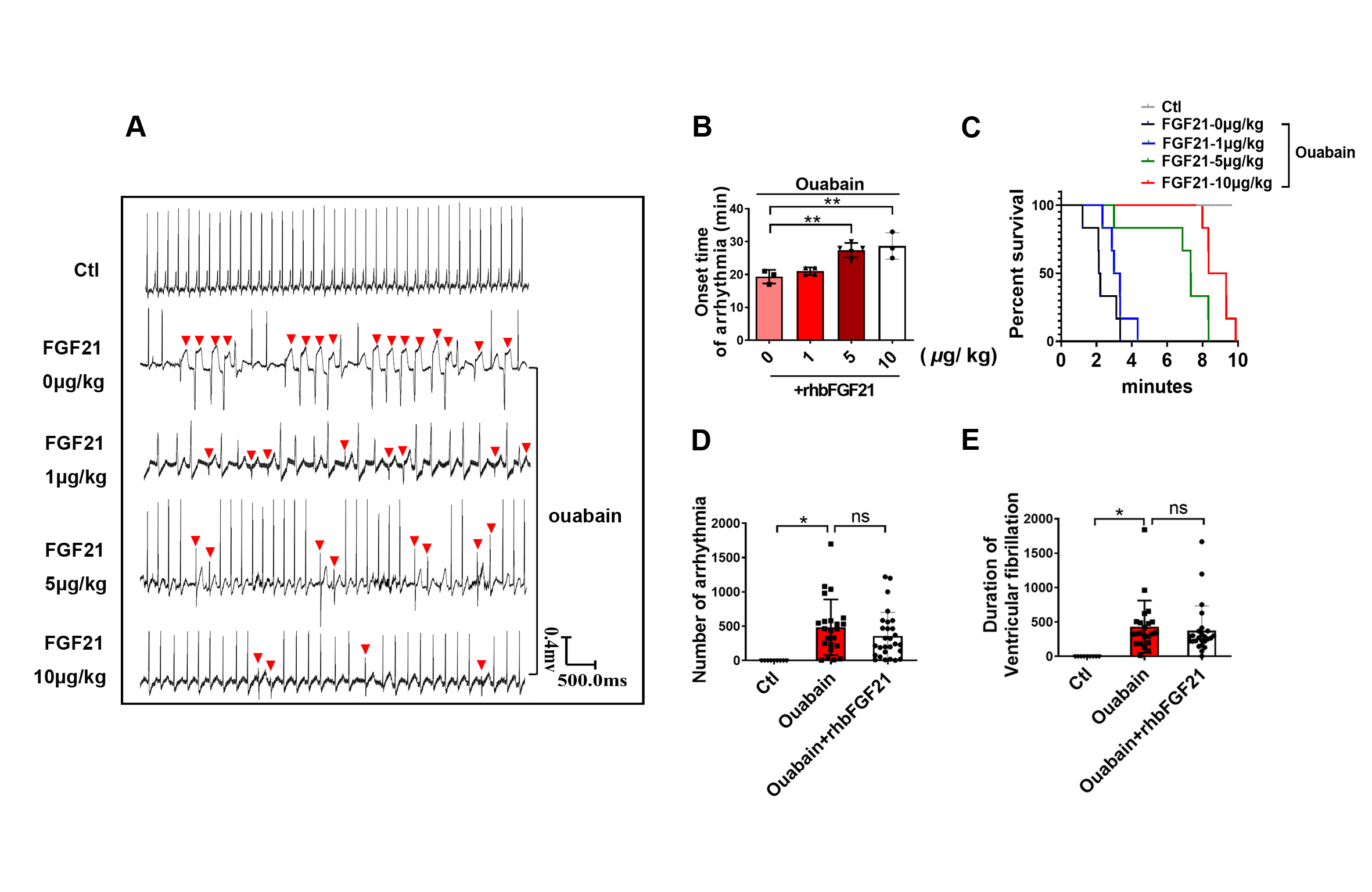

Supplement: Supplementary file 1 [file Image1.TIF]
